# Psychological and Socioeconomic Determinants of Mental Health in Higher Education Students: A Scoping Review

**DOI:** 10.3390/healthcare14121708

**Published:** 2026-06-15

**Authors:** Nazym Zhumagulova, Alla Mireeva, Sholpan Akhelova, Gaukhar Koshkimbayeva, Aizada Askarova, Mariam Taipova, Akerke Amirkhanova, Elmira Kartbayeva, Balzhan Kudaibergenova, Yerbol Kosherbekov, Zukhra Davletgildeyeva, Kenzhebek Bizhanov, Anara Daniyarova, Zhanara Buribayeva

**Affiliations:** 1 Department of Science and Consulting, Kazakhstan Medical University “Higher School of Public Health”, Almaty 050044, Kazakhstan; 2 Department of Public Health, S.D. Asfendiyarov Kazakh National Medical University, Almaty 050012, Kazakhstan; 3 Department of Pharmaceutical Disciplines, Astana Medical University, Astana 010000, Kazakhstan; 4 Department of General Medical Practice with Courses, Kazakh-Russian Medical University, Almaty 050060, Kazakhstan; 5 The Laboratory for the Development of the System of Psychological and Pedagogical Support for Children, National Scientific and Practical Institute for Child Well-Being “Өrken”, Ministry of Education of the Republic of Kazakhstan, Almaty 050000, Kazakhstan; 6 School of Pharmacy, S.D. Asfendiyarov Kazakh National Medical University, Tole-bi 94, Almaty 050012, Kazakhstan; 7 Faculty of Medicine and Healthcare, Farabi University, 71 Al-Farabi Ave., Almaty 050040, Kazakhstan; 8 Sema Hospital, Dostar Med Medical Center, Almaty 050000, Kazakhstan; 9 Department of General Medical Practice No. 2, School of Medicine, S.D. Asfendiyarov Kazakh National Medical University, Almaty 050012, Kazakhstankenzhebek.bizhanov07@gmail.com (K.B.); 10 Department of Interventional Cardiology, Arrhythmology, and Endovascular Surgery, National Scientific Center of Surgery Named After A.N. Syzganov, Almaty 050004, Kazakhstan; 11 Faculty of Medicine and Health Care, Farabi University, Almaty 050040, Kazakhstan; 12 Department of Epidemiology and Biostatistics, Farabi University, Almaty 050040, Kazakhstan; daniyarova.anara@gmail.com; 13 Department of Internal Medicine, S.D. Asfendiyarov Kazakh National Medical University, Almaty 050012, Kazakhstan; 14 Department of Nursing, School of General Medicine No. 1, S.D. Asfendiyarov Kazakh National Medical University, Tole-bi 94, Almaty 050012, Kazakhstan

**Keywords:** mental health, higher education students, psychological determinants, socioeconomic determinants, resilience, coping strategies, financial stress, social support

## Abstract

**Highlights:**

**What are the main findings?**

- Socioeconomic disadvantage, academic pressure, and reduced social support were consistently associated with higher levels of anxiety, depression, and psychological distress among university students.
- Protective factors (e.g., resilience, perceived social support, and adaptive coping strategies) were associated with better mental health outcomes across diverse student populations.

**What are the implications of the main findings?**

- Universities should integrate targeted mental health interventions with academic, financial, and social support services to address key determinants of student distress.
- Early screening and prevention strategies focusing on vulnerable student groups may improve well-being, academic performance, and retention.

**Abstract:**

Background/Objectives: Mental health problems among university students represent a growing public health concern and are shaped by both psychological and socioeconomic determinants that may act independently and interactively. This systematic review aimed to evaluate the separate and combined effects of these determinants on depression, anxiety, stress, and psychological distress in higher education students. Methods: A structured and targeted search strategy using predefined keyword groups and Boolean combinations across PubMed, Scopus, Web of Science, and Google Scholar identified 99 records, of which 19 duplicates were removed. After screening 80 titles and 52 abstracts, 34 full-text articles were assessed for eligibility, and 30 studies were ultimately included in the final review. Data were extracted on study characteristics, mental health outcomes, psychological determinants, socioeconomic factors, and their interactions. Results: The included studies consistently showed that psychological factors, including resilience, coping strategies, loneliness, self-efficacy, and perceived control, were associated with mental health outcomes, with higher resilience and self-efficacy linked to lower levels of depression and anxiety, and maladaptive coping and loneliness associated with increased psychological distress. Socioeconomic determinants, including financial stress, low socioeconomic status, parental education, housing insecurity, and food insecurity also independently contributed to elevated risks of depression, anxiety, and stress. Importantly, several studies demonstrated an interaction between these domains, where socioeconomic disadvantage amplified the adverse effects of poor coping capacity, low resilience, and social isolation, whereas social support and adaptive coping mitigated these effects. Conclusions: Student mental health is influenced by both distinct and interacting psychological and socioeconomic mechanisms, emphasizing the need for integrated institutional strategies that address structural vulnerabilities alongside individual psychological resilience.

## 1. Introduction

Mental health among higher education students is increasingly recognized as a critical public health priority due to its profound influence on student well-being, academic functioning, and long-term life trajectories. A growing body of research shows that a considerable proportion of university students experience psychological difficulties during their studies. Estimates suggest that between 12% and 50% encounter at least one mental health problem, most commonly anxiety, depression, or stress, which can substantially impair both academic performance and everyday functioning [1,2]. Evidence from large international surveys further indicates that approximately 31.4% of first-year students screen positive for at least one common mental disorder within a 12-month period, a prevalence that exceeds that observed in the general population, where pooled global estimates are approximately 17.6%, with higher estimates reported in some national surveys (e.g., 26.2% in the United States) [3,4]. This heightened vulnerability is closely linked to the transitional nature of the higher education period, during which students are simultaneously exposed to multiple stressors, including academic demands, increasing independence, social adaptation, and uncertainty regarding future careers, all of which may exacerbate pre-existing conditions or contribute to the onset of new mental health difficulties [1,2]. Consistent findings across regions confirm the global scope of this issue, with studies reporting high rates of depression and anxiety among students in North America, Europe, Africa, and other regions, and suggesting that approximately 20% of students develop a mental disorder within their first year of study [5]. Notably, recent evidence suggests that these challenges have intensified in the context of the COVID-19 pandemic, which has introduced additional stressors, including social isolation and disruptions to academic routines [1,6,7]. Taken together, these findings indicate that mental health problems in this population are not isolated or transient but are associated with serious consequences, including academic failure, dropout, substance misuse, and increased risk of self-harm and suicide [8,9,10].

Mental health among students cannot be adequately captured by prevalence estimates of depression, anxiety, or other individual symptoms alone, as it is shaped by a complex interaction of psychological, social, and contextual influences. Rather than reflecting only the presence or absence of clinical distress, student mental health is multidimensional and closely linked to both internal psychological resources and the external conditions in which students study and live [11,12]. Existing evidence shows that academic stress is one of the most frequent contributors to mental health deterioration, while disrupted sleep, prolonged use of electronic devices, social isolation, pre-existing conditions, family dynamics, and the educational environment also play important roles in shaping mental well-being [11,12,13]. These observations suggest that psychological factors influence how students perceive and manage adversity, whereas contextual conditions determine the availability of support, stability, and opportunities. Mental health should therefore be viewed not only as a clinical issue but also as a psychosocial one, arising from the interaction between individual vulnerability and the broader educational and social environment [14]. Importantly, this perspective shifts the focus from isolated symptom-based assessment toward a more integrated understanding of student well-being. This is particularly relevant given that mental health difficulties may interfere with students’ ability to continue their studies and engage fully with academic and social life [15,16].

Mental health outcomes among university students are influenced by specific psychological determinants that shape how individuals respond to stress. Factors, including coping strategies, resilience, self-efficacy, and perceived control, influence how students interpret and manage academic and social demands. Adaptive patterns are associated with lower levels of depression, anxiety, and stress, whereas maladaptive coping and poor emotional regulation are linked to increased psychological distress and emotional exhaustion. In addition, loneliness and reduced social connectedness represent important risk factors, particularly among students separated from familiar support systems [17]. Conversely, supportive environments and higher levels of perceived social support are associated with improved mental health outcomes and enhanced psychological functioning [18], consistent with theoretical models emphasizing the role of positive emotional processes in adaptive capacity over time [17].

Socioeconomic conditions play a central role in shaping the environments in which students study, cope with stress, and maintain their mental health. Financial strain, family socioeconomic status, food insecurity, and housing instability can directly affect students’ sense of security and ability to engage with academic demands. Evidence on social determinants of mental health shows that income, education, and access to resources are closely linked to psychological outcomes, with greater socioeconomic disadvantage associated with a higher burden of distress [19,20,21]. In higher education, these inequalities are reflected in students’ daily experiences, including financial strain, limited access to institutional support, unequal availability of resources, and, in some cases, the need to combine study with paid work. Such conditions increase stress exposure and constrain students’ ability to cope effectively. Importantly, social determinants are shaped by broader systems of resource distribution, contributing to persistent mental health inequalities [19]. Addressing these factors is therefore a key target for prevention and public mental health interventions [22]. As a result, differences in student mental health often reflect underlying socioeconomic disparities, highlighting the importance of considering structural alongside individual factors in higher education.

A critical aspect of current research on health inequalities is the recognition that psychological and socioeconomic determinants do not operate separately but interact across multiple levels. Low socioeconomic status is associated with greater exposure to harsh and unpredictable environments, including economic worries, poor housing conditions, and job insecurity, all of which increase stress burden [23,24,25,26,27]. In turn, chronic stress has been linked to impaired executive functioning, reduced self-control, and more short-sighted decision-making, suggesting that financial hardship may weaken coping capacity and shift behavior toward immediate relief rather than long-term benefit [28]. At the same time, supportive social relationships may buffer these adverse effects, as social support has been shown to play a protective role in the association between socioeconomic disadvantage, stress, and health [29]. However, resilience and support should not be interpreted as complete counterbalances to structural disadvantage, because individuals remain embedded in broader socioeconomic conditions that shape their exposure to risk and access to opportunity [30]. Taken together, these observations suggest that socioeconomic adversity and psychological vulnerability reinforce one another, helping to explain the persistence of health inequalities across populations.

Despite the growing body of research on socioeconomic disparities and psychological determinants of health, the literature remains fragmented and lacks integrative synthesis. Many studies examine either socioeconomic factors or psychological processes in isolation, rather than as interacting systems [31,32]. Consequently, current evidence provides only a partial understanding of how these determinants jointly shape mental health outcomes, particularly among students. Findings are also highly heterogeneous due to differences in study design, measurement approaches, and outcome definitions [33]. Moreover, longitudinal evidence capturing dynamic interactions over time is limited, and integration across disciplines remains insufficient [34]. Thus, despite the large volume of research, there is still no coherent map of evidence explaining how socioeconomic and psychological factors interact, highlighting a key gap that this review aims to address.

Given the breadth, heterogeneity, and conceptual fragmentation of the existing literature, a scoping review represents the most appropriate methodological approach for the present study. Scoping reviews are particularly suited to areas where evidence is diverse in terms of study design, populations, and measured outcomes, and where key concepts, including the operationalization of socioeconomic status (e.g., income, parental education, food insecurity), the definition of mental health outcomes (e.g., distress, wellbeing, depression), and the conceptualization of psychological determinants (e.g., stress, coping, self-efficacy), remain inconsistently defined and not systematically integrated across studies. In this context, the objective is not to provide a precise quantitative synthesis of effect sizes, but rather to map the range of socioeconomic and psychological determinants that have been examined, identify how these factors are conceptualized, and explore how their interactions are addressed across studies. This approach also enables the identification of gaps in the literature, including underrepresented populations, methodological limitations, and areas lacking longitudinal or interdisciplinary investigation. Therefore, a scoping review offers a structured and comprehensive framework to organize existing evidence and to clarify the current state of knowledge in a field characterized by complexity and variability.

### Review Questions

This review aims to answer the following questions:

1. What psychological determinants of mental health have been reported in higher education students?
2. What socioeconomic determinants of mental health have been reported in higher education students?
3. How do these determinants interact to influence student mental health?
4. What are the main gaps in the current evidence base?

## 2. Methods

### 2.1. Study Design

This scoping review was conducted and reported in accordance with the Preferred Reporting Items for Systematic Reviews and Meta-Analyses extension for Scoping Reviews (PRISMA-ScR) guidelines (Table S1). The methodological framework proposed by Arksey and O’Malley [35], with further guidance from the Joanna Briggs Institute (JBI) [36], was used to guide the review process. The review was not prospectively registered.

### 2.2. Search Strategy

A comprehensive literature search was conducted across PubMed, Scopus, Web of Science, and Google Scholar using structured keyword-based and Boolean search strategies that combined three main concept blocks: mental health outcomes (e.g., “mental health”, anxiety, depression, stress), population descriptors (e.g., “university students”, “college students”, “higher education”), and determinants (e.g., “socioeconomic”, “financial stress”, income, SES, “social determinants”, “psychological”, coping, resilience, “self-efficacy”). These terms were systematically combined using Boolean operators (AND, OR) to balance sensitivity and specificity; for example, a representative PubMed search string was: (“mental health” OR anxiety OR depression OR stress) AND (“university students” OR “college students” OR “higher education”) AND (“socioeconomic” OR “financial stress” OR income OR SES OR “social determinants”) AND (“psychological” OR coping OR resilience OR “self-efficacy”). Database-specific adaptations were applied, including the use of controlled vocabulary (MeSH terms) in PubMed and field-specific restrictions in Scopus and Web of Science, in accordance with PRESS 2015 guidelines [37]. The literature search was conducted between 15 January 2026 and 25 March 2026, with the most recent search performed on 25 March 2026.

### 2.3. Eligibility Criteria

Studies were considered eligible if they included higher education students (undergraduate or graduate) and examined mental health outcomes, including stress, anxiety, depression, or overall wellbeing. These outcomes were assessed in relation to psychological determinants (e.g., coping strategies, resilience, self-efficacy, emotional regulation, loneliness) and/or socioeconomic determinants (e.g., socioeconomic status, financial stress, income, food insecurity, social support, or parental education). Studies conducted in any geographic or educational setting within higher education were included. A broad range of study designs was considered, including quantitative, qualitative, and mixed-methods studies, as well as relevant review articles published in peer-reviewed journals. Only studies published between 2009 and 2025 were included. No language restrictions were applied. Studies were excluded if they focused exclusively on clinical populations with pre-existing diagnosed mental disorders or examined only biological or genetic factors without consideration of psychological or socioeconomic variables. The included population primarily comprised higher education students, with a predominance of undergraduate cohorts. Although full-time enrollment is typical in this population, detailed reporting of enrollment status, residential conditions (e.g., on-campus vs. off-campus living), and employment participation was not consistently available across the included studies.

### 2.4. Study Selection and Data Extraction

All records identified through the database search were imported into EndNote 2025 (Clarivate Analytics), and duplicates were removed prior to screening. The study selection process was conducted in two sequential stages. First, titles and abstracts were independently screened against the predefined eligibility criteria to identify potentially relevant studies. Second, full-text articles of all selected records were assessed for inclusion. Discrepancies at any stage were resolved through discussion and consensus. No formal quality appraisal was performed in line with scoping review methodology. Studies lacking sufficient methodological detail or relevant outcome data were excluded during screening. A standardized data extraction form was developed to ensure consistency in capturing relevant information across studies. Extracted data included study characteristics (authors, year of publication, country), study design, sample size and population characteristics, mental health outcomes assessed, psychological determinants (e.g., coping strategies, resilience, self-efficacy, emotional regulation, loneliness), socioeconomic determinants (e.g., socioeconomic status, financial stress, social support), and key findings related to the interaction between these factors. The extraction process was iterative, allowing refinement of categories as new themes emerged. This approach enabled a comprehensive mapping of the evidence and facilitated the identification of patterns, relationships, and gaps within the literature.

## 3. Results

### 3.1. Search Results and Study Selection

**Figure 1:**
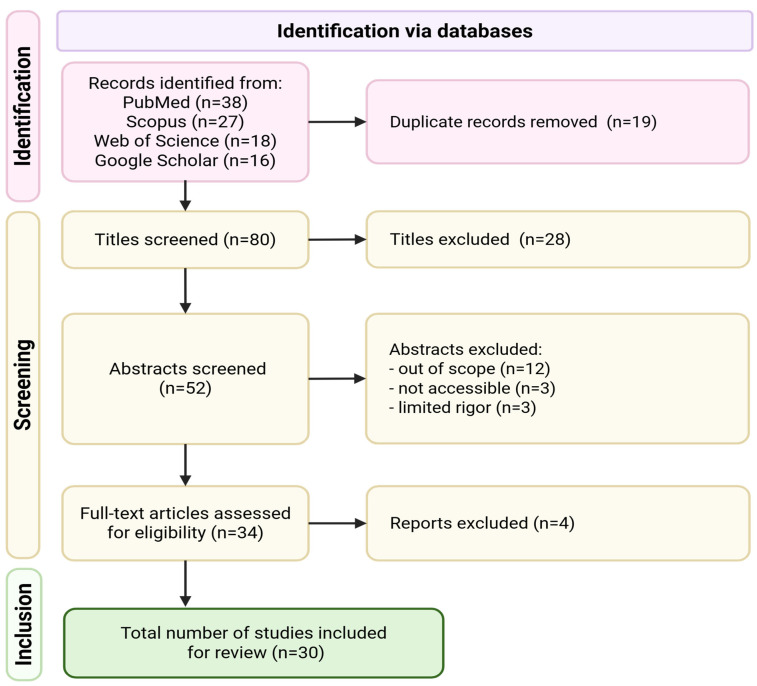
PRISMA-ScR flow diagram of study selection process. Arrows indicate the direction of study selection and screening flow, while colors are used to visually distinguish the identification, screening, and inclusion phases of the review process.

A total of 99 records were identified through database searching (Table S2). After removal of 19 duplicates, 80 records were screened by title, of which 52 were assessed at the abstract level. At the abstract screening stage, records were excluded due to being outside the scope of the review, lack of accessibility, or insufficient methodological detail and relevance to the research question, particularly when studies did not examine psychological or socioeconomic determinants of mental health outcomes. Following full-text evaluation of 34 articles, 30 studies met the inclusion criteria and were included in the final analysis (Figure 1). At the full-text stage, four reports were excluded because they did not provide relevant data on determinants of mental health, focused primarily on intervention or treatment effects without analysis of underlying determinants, or lacked sufficient detail on population characteristics or outcome measures required for inclusion.

### 3.2. Study Characteristics

The included studies (*n* = 30) spanned a wide range of geographical regions, including Europe, Asia, North and South America, as well as multi-country and global analyses, and were predominantly cross-sectional in design, with fewer longitudinal, qualitative, and review-based approaches, reflecting the predominance of observational evidence in the field [38,39,40,41,42,43,44,45,46,47,48,49,50,51,52,53,54,55,56,57,58,59,60,61,62,63,64,65,66,67]. Sample sizes varied substantially, ranging from small qualitative cohorts to large-scale surveys involving several thousand participants, and the populations primarily consisted of undergraduate and graduate students from diverse academic disciplines, including health sciences, social sciences, engineering, and multidisciplinary programs. The included population consisted predominantly of undergraduate students across diverse academic disciplines, as reported in multiple studies [43,45,49,61,62,65], with several focusing specifically on first-year cohorts [41,64] or broader college student populations [50]. While full-time enrollment was typical for these cohorts, detailed information on residential status (e.g., on-campus vs. off-campus living) and employment participation was inconsistently reported, and quantitative data on the proportion of full-time students and residential conditions were not systematically available, limiting precise characterization of these factors. Employment-related variables were not consistently examined across the included studies and did not emerge as primary determinants of mental health outcomes. Only a limited number of studies incorporated employment within broader socioeconomic contexts. For instance, Brito et al. [46] included employment as part of students’ living conditions and financial arrangements, Becerra and Becerra [51] considered employment alongside food insecurity and socioeconomic vulnerability, and Pinho et al. [62] included employment among several contextual variables related to academic and pandemic-related conditions without identifying it as an independent predictor. Collectively, these findings indicate that employment in student populations is better interpreted as a proxy for financial pressure or resource constraints rather than a standalone determinant of mental health. Mental health outcomes were most commonly assessed using validated instruments, including the DASS-21, PHQ-9, GAD-7, Kessler scales (K6/K10), and GHQ-12. Some studies also relied on self-reported or qualitative assessments. The most frequently examined outcomes included depression, anxiety, stress, psychological distress, and overall well-being. Across studies, a broad range of psychological determinants was identified, including coping strategies, resilience, self-efficacy, perceived control, loneliness, and emotional regulation. Socioeconomic determinants, including financial stress, socioeconomic status, parental education, housing conditions, and food insecurity, were consistently reported as key factors influencing mental health outcomes, whereas employment-related variables were not uniformly assessed and appeared only in a subset of studies as part of broader socioeconomic conditions. Only a subset of studies explicitly examined the interaction between psychological and socioeconomic determinants, typically through mediation or moderation analyses, underscoring the complex and interdependent nature of these influences.

### 3.3. Psychological Determinants of Mental Health

Across the included studies, psychological determinants were strongly associated with mental health outcomes, with perceived stress and academic pressure consistently showing the largest effects (Table 1). For example, perceived stress demonstrated a strong association with depression in longitudinal modelling (β = 0.57) [67], while academic pressure and stress were among the most frequently reported predictors of anxiety and depression in a large-scale global synthesis of evidence [52], with prevalence estimates reaching 23–48% for depression and 21–36% for anxiety. In individual studies, academic stress was also a significant predictor of mental health outcomes (β = 0.39) [58], confirming its consistent impact across different populations. Protective psychological factors showed smaller but consistent effects. Perceived control and self-efficacy acted as mediators of mental health outcomes, with socioeconomic status indirectly influencing wellbeing through these variables (r = 0.30 for positive wellbeing; r = −0.16 for negative wellbeing) [39]. In contrast, loneliness and low self-esteem were moderately associated with symptom severity, with correlation coefficients reaching rs ≈ 0.49–0.52 [59], while food insecurity-related distress (closely linked with psychological vulnerability) showed strong associations (OR = 3.65) [51]. Less frequently reported mechanisms, including minority stress and identity-related strain, were identified in qualitative analyses [42], indicating additional high-risk pathways in specific populations. Overall, the findings demonstrate that stress-related processes represent the strongest and most consistent psychological drivers of mental health outcomes, whereas regulatory and social-cognitive factors act primarily as modifiers of these effects.

### 3.4. Socioeconomic Determinants of Mental Health

Socioeconomic determinants were consistently associated with mental health outcomes, with financial stress, low socioeconomic status, and resource insecurity emerging as the most robust and frequently reported risk factors across studies. Financial stress showed strong effects in individual analyses (β = 0.45) [47], while food insecurity was associated with substantially increased odds of psychological distress (OR = 3.65) [51]. Similarly, lower socioeconomic status was linked to poorer mental health outcomes, including reduced wellbeing (r = 0.30) and increased negative mental health indicators (r = −0.16) [39], confirming consistent associations across different analytical approaches. Evidence from a large-scale global synthesis [52] further supports these findings, with financial strain, housing instability, and inequality repeatedly identified as key contributors to depression, anxiety, and stress, with prevalence estimates reaching up to 48% for depression and 36% for anxiety in socioeconomically vulnerable groups.

In addition to direct effects, socioeconomic conditions also shaped mental health through structural and contextual mechanisms. Lower income, parental education, and social vulnerability were consistently associated with higher levels of distress and academic difficulties, with some studies reporting strong associations with adverse outcomes (OR up to 3.99) [48]. Deprivation and lower family affluence also increased the risk of depression and reduced protective factors, including perceived control [49]. Broader contextual factors, including living conditions, and institutional resources, further influenced mental health, particularly during periods of instability, including the COVID-19 pandemic [57,60,62]. Overall, the evidence indicates that socioeconomic determinants act as both direct risk factors and structural drivers of mental health inequalities, shaping exposure to stressors and access to protective resources.

### 3.5. Interaction Between Psychological and Socioeconomic Determinants

The interaction between socioeconomic and psychological determinants across the included studies follows a consistent and structured pattern in which socioeconomic disadvantage primarily exerts its effects on mental health through stress-related and cognitive–emotional pathways, rather than acting as an isolated factor. Financial strain, low socioeconomic status, and resource insecurity were repeatedly associated with elevated levels of perceived stress, which in turn demonstrated the strongest and most consistent relationship with depression, anxiety, and psychological distress, including large effect sizes observed in longitudinal modelling (β = 0.57) [67] and strong associations with financial stress (β = 0.45) [47] and food insecurity (OR = 3.65) [51]. This indicates that socioeconomic conditions operate as upstream determinants that increase exposure to stressors, while psychological processes determine how these stressors are internalized and translated into mental health outcomes. Beyond stress-driven pathways, the interaction is further reinforced by resource-based mechanisms, whereby socioeconomic position shapes access to psychological assets such as control, support, and coping capacity. Importantly, these mechanisms do not operate independently but interact dynamically, amplifying vulnerability under conditions of cumulative disadvantage. At the same time, this interaction is not unidirectional, as psychological resources also modify the strength of socioeconomic effects: higher levels of resilience, adaptive coping, and perceived social support attenuate the impact of socioeconomic disadvantage, although these buffering effects remain partial, given that low social support can increase the risk of mental health problems by up to fivefold [53]. Importantly, the interaction becomes more pronounced in contexts of overlapping vulnerability, where socioeconomic disadvantage co-occurs with identity-related and environmental stressors, including minority status, institutional exclusion, or unstable living conditions. This co-occurrence leads to a compounded psychological burden that is not fully captured by linear models [42]. Hence, the evidence supports a multidimensional interaction model in which socioeconomic factors shape both exposure to stress and access to resources, psychological determinants regulate individual response, and their combined effects produce cumulative and reinforcing impacts on student mental health.

Evidence from quantitative studies consistently indicates that the relationship between SES and mental health outcomes among university students is not direct but operates through multiple interconnected psychological mechanisms. Structural equation modelling in a large UK sample (*n* = 811) demonstrated that SES was significantly associated with both positive (β = 0.271) and negative wellbeing (β = −0.145), with indirect effects primarily mediated by perceived control (β = 0.085; β = −0.055) and competence (β = 0.045; β = −0.051), while inclusion contributed only to positive wellbeing (β = 0.059) and perceived worth showed no independent effect [39]. Similar patterns were observed in a Chinese cohort during COVID-19 lockdown (*n* = 839), where SES was negatively associated with psychological distress (β = −0.119) and loneliness (β = −0.132), and positively associated with life satisfaction (β = 0.090), with both parallel and sequential mediation effects identified through perceived social support (β range −0.021 to 0.043) and self-efficacy (β range −0.020 to 0.060), including significant chained pathways (β range −0.011 to 0.033) [44]. Earlier regression-based evidence further supports partial mediation, showing that the protective effect of higher SES on depressive symptoms (OR ≈ 0.3–0.5) was reduced by approximately 52% after inclusion of perceived control, which itself was strongly associated with depression risk (OR = 1.6) [49]. More complex longitudinal modelling confirms the presence of multi-level pathways, where perceived stress emerged as the strongest direct predictor of depression (β = 0.57), while self-esteem exerted a protective effect (β = −0.57), and indirect effects were transmitted through sequential mechanisms involving coping and behavioural factors (e.g., self-esteem β = −0.31; negative coping β = 0.26) [67]. Collectively, these findings demonstrate that SES-related disparities in mental health are shaped by interacting psychological processes operating through parallel and sequential pathways, rather than a single direct association.

**Figure 2:**
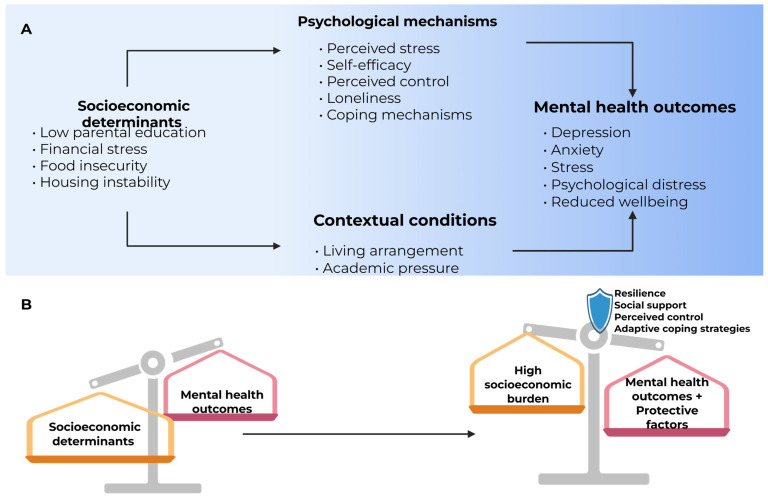
Conceptual model of the interaction between socioeconomic and psychological determinants of mental health outcomes among higher education students: (**A**) Schematic representation of the primary pathways identified across the included studies, illustrating how socioeconomic determinants act as upstream drivers influencing mental health outcomes through psychological mechanisms. In this framework, factors such as financial stress, low socioeconomic status, and resource insecurity increase exposure to stressors, while psychological processes including perceived stress, self-efficacy, perceived control, loneliness, and coping mechanisms mediate and regulate individual responses, ultimately shaping mental health outcomes. Contextual conditions, such as living arrangements and academic pressure, further contribute to this process by influencing both exposure and outcome levels. (**B**) Conceptual illustration of the balance between accumulated risk and protective factors. In this model, protective factors, including resilience, social support, perceived control, and adaptive coping strategies, attenuate the negative impact of socioeconomic disadvantage and psychological stress. Although these factors may not fully eliminate the effects of structural risk, they contribute to improved mental health outcomes by partially buffering adverse effects and promoting greater psychological stability.

The interaction between socioeconomic disadvantage and psychological mechanisms shapes mental health outcomes through stress-related pathways and partial buffering by protective factors (Figure 2). Conceptually, this relationship is best understood as a dynamic and non-linear system in which exposure, regulation, and context operate simultaneously rather than sequentially. Socioeconomic conditions do not act in isolation but define the intensity and persistence of stress exposure, while psychological processes determine how these conditions are interpreted and managed at the individual level. Importantly, variability in outcomes across student populations reflects differences in regulatory capacity and access to supportive resources, rather than uniform effects of exposure. This perspective emphasizes that mental health disparities arise from the combined influence of structural constraints and individual response mechanisms, highlighting the need to consider both domains within an integrated framework.

## 4. Discussion

### 4.1. Summary of Key Findings

Across the 30 included studies, the evidence reveals a consistent structural pattern in which mental health outcomes are shaped by the joint distribution of psychological vulnerability and socioeconomic position, rather than by isolated effects of individual variables. Notably, a substantial proportion of the included studies, primarily cross-sectional in design (e.g., [39,41,43,44,46,47,48,51,57,58,59,60,61,62,65]), were conducted across diverse geographical contexts, yet they converged on similar patterns of high prevalence of anxiety, depression, and stress. This consistency suggests that these outcomes are not context-specific but broadly reproducible across populations. In addition, the findings highlight that variability in mental health outcomes is not primarily explained by differences in exposure alone, but by differences in how students respond to comparable conditions, as reflected in the repeated identification of heterogeneity across subgroups defined by gender, academic field, socioeconomic background, and living conditions [43,57,60,62]. Furthermore, the evidence suggests that psychological and socioeconomic determinants cluster together, with financial strain frequently co-occurring with academic pressure, social isolation, and reduced access to support, forming cumulative risk profiles rather than independent predictors [40,52,55]. At the same time, longitudinal designs were limited to a small number of studies (e.g., [50,67]), and only a subset explicitly modelled interaction or mediation pathways (e.g., [39,44,45,67]), indicating that the available evidence predominantly captures static associations rather than dynamic processes, thereby constraining the ability to fully understand how these determinants evolve over time. Taken together, these findings suggest that student mental health is characterized by reproducible patterns of risk, subgroup variability, and clustered exposures, supporting a systemic interpretation in which both structural conditions and individual responses jointly determine outcomes.

### 4.2. Interpretation of Psychological Determinants

Psychological determinants in the included evidence are best interpreted not as isolated traits, but as mechanisms that regulate how students process external stress, because the same pandemic context produced markedly different mental health outcomes depending on stress appraisal, coping capacity, and internal psychological resources. This is especially clear in studies showing that perceived stress, fear, uncertainty, and emotional dysregulation were repeatedly linked to anxiety, depression, and distress, including high symptom burdens in large student cohorts [68,69,70], whereas reduced emotional regulatory self-efficacy was associated with depressive symptoms in a very large Chinese sample [71]. At the same time, the evidence does not support a purely deficit-based interpretation, because meaning in life, resilience, mindfulness, and spirituality repeatedly appeared as factors associated with lower distress or more favorable psychological functioning [72,73,74], suggesting that psychological determinants act bidirectionally, as both vulnerability pathways and compensatory resources. However, this literature also has important weaknesses. Cross-sectional self-report designs predominated among the included studies (e.g., [39,41,43,44,46,47,48,51,57,58,59,60,61,62,65]), which limit causal inference and make it difficult to determine whether factors, including low self-efficacy, loneliness, or maladaptive coping, precede distress or are themselves consequences of it. Moreover, the evidence is uneven in quality, as some findings derive from very large surveys, whereas others come from small or highly specific subgroups, including first-year medical students or LGBT students [75,76]. Taken together, the available evidence supports the interpretation that psychological determinants function primarily as a processing system through which crisis-related stressors are amplified, contained, or transformed, but the predominance of observational designs means that their causal hierarchy remains only partially resolved.

### 4.3. Interpretation of Socioeconomic Determinants

Socioeconomic determinants in the included evidence function not merely as background variables but as structural conditions that shape both exposure to stressors and access to coping resources, thereby exerting direct and indirect effects on student mental health. Evidence from large-scale pandemic studies demonstrates that concrete material constraints, including financial hardship, difficulty accessing basic needs, and unstable living conditions, are consistently associated with increased anxiety and distress through disruptions in daily functioning and academic engagement [70,77]. Population-level data further indicate a substantial mental health burden under conditions of socioeconomic disruption, with prevalence estimates of approximately 45% for overall mental health problems and 34.9%, 21.1%, and 11.0% for acute stress, depression, and anxiety, respectively [68]. These patterns are reinforced by findings identifying specific structural exposures, including rural or peripheral residence, low income, and infection risk within social networks, as significant predictors of psychological distress [78,79]. Importantly, the evidence synthesized across the 30 included studies provides convergent and quantitatively grounded support for these associations, demonstrating that financial stress (β = 0.45) [47], food insecurity (OR = 3.65) [51], and broader social vulnerability (OR up to 3.99) [48] are among the strongest predictors of adverse mental health outcomes, while socioeconomic gradients are further reflected in correlations with wellbeing (r = 0.30; r = −0.16) [39] and increased depressive symptom burden under conditions of deprivation [49]. Moreover, socioeconomic influences extend beyond income-based indicators, encompassing parental education, housing conditions, and access to institutional and social resources [43,44,50,60,62,65], thereby supporting a multidimensional model of socioeconomic constraint. At the same time, heterogeneity across studies indicates that these effects are context-dependent, as some cohorts report stable or even reduced distress under comparable macro-level conditions [80,81], likely reflecting differences in institutional support, living environments, and resource availability [82].

Across the included studies, contextual variables such as residence, enrollment status, and macro-level economic setting were unevenly reported but reveal several structured patterns. With regard to living arrangements, multiple studies explicitly incorporated residence-related factors, including living conditions, housing stability, and whether students resided with family or independently (e.g., [38,43,48,60,61,62,65]). These factors were frequently reported in association with mental health outcomes, with unstable or resource-limited living environments linked to higher levels of distress, anxiety, and depression, particularly in the context of financial strain and reduced access to support systems [48,60]. In contrast, enrollment-related variables, including full-time versus part-time status, were rarely examined directly, with only indirect proxies such as academic workload, year of study, or performance used to approximate differences in student engagement (e.g., [43,50,58,62]), limiting the ability to draw conclusions regarding their independent effects. At the macro level, the included studies span both high-income countries (HICs), including the United Kingdom, USA, France, Belgium, Portugal, Spain, Sweden, Germany, Croatia, and Serbia [39,41,45,51,57,60,63], and low- and middle-income countries (LMICs), including Bangladesh, India, Saudi Arabia, Brazil, Malaysia, and China [38,40,42,43,44,46,47,48,50,58]. Despite this diversity, the overall pattern of associations between socioeconomic disadvantage and adverse mental health outcomes was consistent across settings, although studies conducted in LMICs more frequently emphasized material constraints, including financial hardship, food insecurity, and limited institutional resources, as primary drivers of distress [40,47,48], whereas studies from HICs more often highlighted psychosocial mechanisms, including perceived control, social support, and coping processes [39,41,45]. However, the lack of standardized reporting and limited use of stratified or comparative analyses across these contextual dimensions constrain the ability to systematically evaluate their moderating effects.

A critical limitation lies in the inconsistent operationalization of socioeconomic variables, which are frequently measured through proxies, including residence, parental background, or indirect indicators of resource access [41,49,50], rather than standardized SES constructs. This limits comparability and may lead to an underestimation of structural effects. Taken together, the integrated evidence supports a system-level interpretation in which socioeconomic determinants act as primary upstream drivers of differential exposure to risk, shaping both the intensity and persistence of psychological distress, while interacting with contextual and institutional factors that remain insufficiently captured in current research.

### 4.4. Integrated Perspective

Taken together, the evidence supports a system-level interpretation in which student mental health arises from the interaction between structurally determined exposure and psychologically mediated response, rather than from isolated determinants. Across the included studies, socioeconomic conditions consistently shape exposure to stressors by influencing financial stability, living arrangements, and access to academic and institutional resources [43,44,50,60,62]. Psychological factors regulate how these conditions are experienced and translated into outcomes through mechanisms, including perceived control, coping, and social support [39,47,55,61]. Within this integrated framework, stress-related processes represent a central pathway linking external pressures to mental health outcomes, as both socioeconomic adversity and academic disruption converge on elevated stress, anxiety, and depressive symptoms across diverse contexts [52,53]. However, the relationship is not linear, because individual differences in regulatory capacity explain why similar levels of exposure lead to heterogeneous outcomes, with resilience and social support partially buffering adverse effects but not fully offsetting structural disadvantage [39,58]. This asymmetry suggests that psychological resources operate within constraints imposed by socioeconomic conditions, rather than independently of them. Furthermore, variation across studies indicates that contextual factors, including institutional support, learning environments, and broader social conditions, further modulate this interaction [60,62,65], reinforcing the view that mental health outcomes reflect a multilevel system linking structural inequality, individual regulation, and environmental context. Collectively, these findings support a shift from linear cause–effect models toward an integrated framework in which exposure, psychological processing, and context interact to determine both the severity and persistence of psychological distress.

### 4.5. Gaps in the Literature

Despite the growing body of evidence, several consistent limitations emerge across the included studies that constrain the strength and interpretability of current findings. First, the predominance of cross-sectional designs [39,43,44,46,47,51,58,61,62,65] limits causal inference, making it difficult to disentangle whether socioeconomic disadvantage and psychological factors act as antecedents or consequences of mental health outcomes, with only a small number of longitudinal analyses available [50,67]. Second, there is substantial heterogeneity in the operationalization of socioeconomic determinants. These variables are frequently measured through indirect proxies, including parental education, residence, or grant status [41,49,50], or through inconsistently defined composite indices [44,58], rather than standardized SES frameworks, which reduces comparability across studies and likely leads to an underestimation of structural effects. Third, interaction and mediation mechanisms between psychological and socioeconomic determinants remain insufficiently examined, as direct association analyses predominated across the included studies (e.g., [38,41,43,46,47,48,50,51,52,53,55,56,57,58,59,60,61,62,65]), while formal mediation or structural equation modelling was applied in only a small subset of studies (e.g., [39,44,45,67]), leaving integrated mechanisms largely inferential rather than empirically validated. Fourth, the evidence base is characterized by sampling and contextual bias, with overrepresentation of specific regions and populations, including high-income settings or single-institution samples [43,46,47], alongside underrepresentation of diverse socioeconomic contexts, which limits generalizability and obscures global variability. Finally, methodological limitations, including reliance on self-reported measures, variability in assessment tools, and inconsistent reporting of effect sizes, further weaken the robustness of conclusions [38,40,54]. Collectively, these gaps indicate that current research remains fragmented and has limited capacity to fully capture the multilevel and interacting nature of psychological and socioeconomic determinants of student mental health.

### 4.6. Implications

The integrated evidence has several important implications for intervention, policy, and institutional practice. First, the consistent role of psychological processes as proximal regulators of mental health suggests that interventions targeting coping strategies, perceived control, and emotional regulation may yield substantial benefits, particularly when implemented at scale within university settings [39,47,55,61]. However, the findings also indicate that such approaches alone are insufficient, as socioeconomic disadvantage, including financial stress, food insecurity, and low socioeconomic status, exerts strong and persistent effects on mental health outcomes [47,48,51,58], implying that effective intervention requires parallel structural strategies addressing material conditions. This highlights the need for integrated models of support in which psychological services are complemented by financial assistance, housing stability programs, and improved access to academic and institutional resources [43,60,62]. Furthermore, the observed interaction between exposure and regulation suggests that early identification of high-risk groups, particularly students experiencing both socioeconomic vulnerability and low coping capacity, may improve targeting and efficiency of interventions. At a broader level, these findings support a shift from individual-level mental health frameworks toward multilevel approaches that incorporate social determinants of health into student support systems, emphasizing that improving mental health outcomes requires not only strengthening individual resilience but also reducing structural inequalities within educational environments.

### 4.7. Limitations

This review has several limitations that should be considered when interpreting the findings. First, the inclusion of studies with heterogeneous designs, populations, and measurement tools introduces variability that may limit the comparability and synthesis of results. Second, although efforts were made to include a broad range of evidence, the review is subject to potential selection bias due to inclusion criteria and database coverage, which may have led to the exclusion of relevant studies. Third, the reliance on published literature raises the possibility of publication bias, as studies reporting significant associations are more likely to be included. Additionally, variability in reporting standards across studies, including inconsistent availability of effect sizes and methodological details, may affect the robustness of the synthesis. Finally, as a predominantly narrative integration of findings, the review does not provide quantitative meta-analytic estimates, which limits the ability to determine the magnitude of pooled effects.

### 4.8. Future Research

Future research should move beyond predominantly cross-sectional and descriptive designs toward longitudinal, multi-wave, and mechanism-oriented studies capable of clarifying the temporal ordering between socioeconomic adversity, psychological regulation, and mental health outcomes, because the current evidence base remains heavily weighted toward one-time assessments and therefore cannot adequately distinguish antecedents from consequences. Greater methodological standardization is also needed, particularly in the measurement of socioeconomic conditions, which in the included studies were often represented by indirect proxies, including parental education, residence, food insecurity, living arrangements, or broad social vulnerability, rather than harmonized multidimensional SES indices [41,44,48,49,50,51,58,60,62]. In addition, future studies should more systematically test mediation, moderation, and interaction pathways, since only a limited subset of the current literature employed structural or sequential models [39,44,45,67], despite repeated indications that psychological and socioeconomic determinants operate jointly rather than independently. The evidence base would also benefit from broader geographic and institutional representation, as both the present review and the scoping review by Dias et al. indicate clustering of studies within a limited number of countries and settings, with uneven coverage of low-resource educational environments and socially marginalized student groups [82]. Finally, future research should prioritize intervention-based and implementation-focused designs that evaluate whether integrated approaches combining mental health support with financial, housing, and academic assistance produce more durable benefits than psychologically focused interventions alone, thereby translating the current descriptive evidence into actionable, equity-oriented models of student support.

## 5. Conclusions

This review provides a comprehensive synthesis of psychological and socioeconomic determinants of mental health among higher education students, demonstrating that these factors operate not as independent predictors but as interconnected components of a multilevel system. Across the included studies, mental health outcomes were consistently shaped by the combined effects of structural exposure and individual psychological regulation, with socioeconomic disadvantage defining the intensity and persistence of stressors, and psychological processes determining how these stressors are internalized and expressed. In particular, stress-related mechanisms emerged as the most consistent pathway linking diverse forms of adversity to depression, anxiety, and psychological distress. Protective factors, including perceived control, resilience, and social support, acted as partial buffers rather than full compensatory mechanisms.

Importantly, the findings highlight that mental health inequalities among students are largely structured by material and contextual conditions, including financial stress, resource insecurity, and unequal access to institutional support, which cannot be adequately addressed through individual-level interventions alone. At the same time, the evidence indicates variability across populations and study contexts, suggesting that outcomes are shaped by the interaction of structural, psychological, and contextual factors rather than by uniform risk patterns. However, the predominance of cross-sectional designs, inconsistent measurement of socioeconomic variables, and limited use of interaction modelling constrain the ability to fully resolve causal pathways and dynamic processes.

Overall, this review supports a shift toward integrated and multilevel approaches to student mental health, emphasizing that effective prevention and intervention strategies must simultaneously address psychological regulation and structural inequality. Advancing this field will require methodologically robust, longitudinal, and intervention-oriented research capable of translating current descriptive evidence into actionable frameworks that improve both equity and mental health outcomes in higher education settings.

## Figures and Tables

**Table 1:** Characteristics of included studies and psychological and socioeconomic determinants of mental health among higher education students.

Author (Year)	Country & Sample	Study Design & Field	Mental Health Outcomes	Psychological Determinants	Socioeconomic Determinants	Interaction/Mediation	Key Findings
Zhu et al. (2022) [50]	China; *n* = 4907 students	Longitudinal; Mixed	Psychological distress	Academic performance	Parental education, family economic condition, residence	None	13.5% increased distress
Tabor et al. (2021) [63]	UK; *n* = 11,519	Longitudinal; General	Distress (GHQ-12)	Baseline MH	Higher education status, parental education as SES proxy	Stratified	Students lower distress
Qing & Yang (2026) [67]	China; *n* = 880 students	Longitudinal SEM; Mixed	Depression	Trauma, stress	Rural background, lower parental education, non-only-child status, left-behind experience, bullying, sexual minority status, family environment	Mediation (SEM)	Stress strongest predictor (β = 0.57)
Dougall et al. (2023) [39]	UK; *n* = 811 students	Cross-sectional SEM; Mixed	Positive/negative wellbeing	Control, competence, inclusion	Subjective SES, parental income, education, occupation, economic and social capital	Mediation	SES linked to wellbeing (r = 0.30; −0.15)
Huang & Wang (2023) [44]	China; *n* = 839 students	Cross-sectional + mediation; Mixed	Distress, loneliness	Social support, self-efficacy	Composite SES based on parental education, occupation, and family wealth	Mediation	Indirect SES effects significant
Fontaine (2021) [45]	USA; *n* = 449 students	Cross-sectional regression; Mixed	Anxiety, depression	Perfectionism, relationships	Family SES, parental education, parental social status, family background	Mediation & interaction	Social support protective
Ibrahim et al. (2013) [49]	UK; *n* = 923 students	Cross-sectional mediation; Mixed	Depression	Sense of control	Family affluence, area deprivation, parental education, parental occupation	Partial mediation	Deprivation ↑ risk
Villatoro et al. (2023) [60]	USA; *n* ≈ 746 students	Cross-sectional; Mixed	Distress (K6)	Help-seeking	Low income, job loss, discrimination, nativity, institutional mental health resources	None	Low income ↑ distress
Monteiro et al. (2024) [58]	Brazil; *n* = 323 students	Cross-sectional; Mixed	Mental health	Academic stress, resilience	Socioeconomic class, time spent at university	None	R^2^ ≈ 48%
Verger et al. (2009) [41]	France; *n* = 1723 students	Cross-sectional; Mixed	Psychological distress	Mastery, social support	Grant status used as SES proxy; field of study; academic context	Stratified	Poor adjustment ↑ distress
Mohammad et al. (2020) [43]	Saudi Arabia; *n* = 373 students	Cross-sectional; Mixed	Depression, anxiety, stress	Academic burden	Income, father’s education, mother’s education, residence, marital status	None	Higher prevalence in low SES
Becerra & Becerra (2020) [51]	USA; *n* = 302 students	Cross-sectional; Mixed	Distress	Physical health, alcohol	Food insecurity, employment, ethnicity, nativity	Stratified	OR = 3.65
Ramón-Arbués et al. (2020) [61]	Spain; *n* = 1074 students	Cross-sectional; Mixed	DASS-21 outcomes	Self-esteem, insomnia	Living arrangement, relationship status, financial status, lifestyle patterns	None	Stress 34%
Schmits et al. (2021) [57]	Belgium; *n* = 23,307 students	Cross-sectional; Mixed	Anxiety, depression	Emotional distress	Financial deterioration and socioeconomic stress during COVID-19	None	Prevalence > 50%
Soares et al. (2012) [59]	Portugal; *n* = 300 students	Cross-sectional; Mixed	Psychological symptoms	Loneliness	Gender, age, year, course, academic performance, parental education, living arrangement	None	r ≈ 0.5
Zakaria et al. (2025) [47]	Malaysia; *n* = 113 students	Cross-sectional descriptive; Mixed	Mental well-being	Social support	Financial stress	None	R^2^ = 56.6%
Dias (2025) [48]	Brazil; service users	Cross-sectional analytical; Mixed	Anxiety, depression	Emotional symptoms	Social vulnerability, living arrangement, family context	None	OR up to 3.99
Brito et al. (2021) [46]	Brazil; *n* = 135 dental students	Cross-sectional; Dentistry	Stress	Coping phases	Financing of education, family support, living conditions, employment, marital status	None	Stress 62%
Ivanović et al. (2025) [65]	Croatia/Serbia; *n* = 424	Cross-sectional; Nursing	Stress, anxiety	Academic stress	Living conditions, family environment, social support, socioeconomic background, academic context	None	Significant differences
Samsonenko (2023) [64]	Russia; *n* = 68 students	Cross-sectional; Pedagogy	Neuro-psych stability	Personality traits	Socioeconomic determinants not central in the study	None	Extraversion protective
Pimienta et al. (2025) [38]	Mexico; ~980 students	Non-experimental; Psychology	Well-being, loneliness	Social skills, resilience	Place of origin, living conditions, community context, privacy limits, social media exposure, local opportunities	None	Descriptive only
Son et al. (2020) [56]	USA; *n* = 195 students	Mixed-methods; Mixed	Stress, anxiety	Fear, sleep disruption	Difficulty accessing services/basic needs may be context-related, but not clearly analyzed as SES	None	71% increased stress
David (2026) [42]	India; *n* = 13 students	Qualitative (IPA); Mixed	Distress, identity	Minority stress	Class, caste, religion, region, hostel surveillance, institutional conservatism	Intersectional	Thematic results
Hasan (2024) [40]	Bangladesh; 24 studies	Systematic review; Mixed	Anxiety, depression	Isolation, smartphone use	Financial hardship, unstable income, internet access and cost, living arrangement, institutional differences	Narrative	High prevalence
Segar & Kosnin (2024) [53]	Global; 18 studies	Systematic review; Mixed	Multiple disorders	Personality, gender	Family economic status, parental education, ethnicity, sexual orientation, educational disruption, financial stress	None	Risk ↑ up to 5-fold
Roy et al. (2025) [52]	Global; >1.2 M	Scoping review; Mixed	Stress, anxiety	Coping, sleep	Financial stress, SES inequality, housing and food insecurity, stigma, service access	Conceptual	20–48% prevalence
Mofatteh (2021) [55]	Multi-country; 41 studies	Narrative review; Mixed	Stress, anxiety	Self-esteem	Low family income, poverty, lack of financial support, minority/international student status	None	6 risk domains
Chemagosi (2024) [54]	Global	Narrative review; Mixed	Distress	Academic pressure	Financial constraints, SES, cultural background, family dynamics, access to resources	Conceptual	No stats
Cant (2018) [66]	UK	Conceptual paper	Distress	Identity strain	Widening participation, class inequality, debt, unequal access to elite education, labor-market congestion	Conceptual	No stats

SES, socioeconomic status; MH, mental health; SEM, structural equation modeling; K6, Kessler Psychological Distress Scale; GHQ-12, 12-item General Health Questionnaire; DASS-21, Depression, Anxiety and Stress Scale-21; IPA, Interpretative Phenomenological Analysis; OR, odds ratio; β, standardized regression co-efficient; R^2^, coefficient of determination; UK, United Kingdom; USA, United States of America, ↑, increased/higher.

## Data Availability

No new data were created or analyzed in this study.

## Supplementary Materials

The following supporting information can be downloaded at: https://www.mdpi.com/article/10.3390/healthcare14121708/s1, Table S1: PRISMA-ScR checklist [83]; Table S2: Search strategy and database queries used for the literature search.

## Abbreviations

The following abbreviations are used in this manuscript:

CRAFFT	Car, Relax, Alone, Forget, Friends, Trouble screening tool
CTQ	Childhood Trauma Questionnaire
DASS-21	Depression Anxiety Stress Scales, 21-item version
GAD-7	Generalized Anxiety Disorder 7-item scale
GHQ-12	General Health Questionnaire, 12-item version
HADS	Hospital Anxiety and Depression Scale
IAT	Internet Addiction Test
IPAQ	International Physical Activity Questionnaire
ISI	Insomnia Severity Index
ISS	Lipp’s Stress Symptoms Inventory
JBI	Joanna Briggs Institute
K6	Kessler Psychological Distress Scale, 6-item version
K10	Kessler Psychological Distress Scale, 10-item version
MHI-5	Mental Health Inventory, 5-item version
OLS	Ordinary Least Squares
PHQ-2	Patient Health Questionnaire, 2-item version
PHQ-9	Patient Health Questionnaire, 9-item version
PRISMA	Preferred Reporting Items for Systematic Reviews and Meta-Analyses
PRISMA-ScR	Preferred Reporting Items for Systematic Reviews and Meta-Analyses extension for Scoping Reviews
PRESS	Peer Review of Electronic Search Strategies
QSG-12	General Health Questionnaire, 12-item version (Portuguese acronym)
RSES	Rosenberg Self-Esteem Scale
SCL-90-R	Symptom Checklist-90-Revised
SDS	Self-Rating Depression Scale
SEM	Structural Equation Modeling
UFV	Federal University of Viçosa
ULS-8	UCLA Loneliness Scale, 8-item version
WHO-5	World Health Organization-Five Well-Being Index
ZDS	Zagazig Depression Scale
